# Early Emergence and Long-Term Persistence of HIV-Infected T-Cell Clones in Children

**DOI:** 10.1128/mBio.00568-21

**Published:** 2021-04-08

**Authors:** Michael J. Bale, Mary Grace Katusiime, Daria Wells, Xiaolin Wu, Jonathan Spindler, Elias K. Halvas, Joshua C. Cyktor, Ann Wiegand, Wei Shao, Mark F. Cotton, Stephen H. Hughes, John W. Mellors, John M. Coffin, Gert U. Van Zyl, Mary F. Kearney

**Affiliations:** a HIV Dynamics and Replication Program, CCR, National Cancer Institute, Frederick, Maryland, USA; b Leidos Biomedical Research, Inc., Frederick National Laboratory for Cancer Research, Frederick, Maryland, USA; c Department of Medicine, University of Pittsburgh, Pittsburgh, Pennsylvania, USA; d Department of Pediatrics and Child Health, Tygerberg Children’s Hospital and Family Center for Research with Ubuntu, Stellenbosch University, Cape Town, South Africa; e Department of Molecular Biology and Microbiology, Tufts University, Boston, Massachusetts, USA; f Division of Medical Virology, Stellenbosch University and National Health Laboratory Service Tygerberg, Cape Town, South Africa; Columbia University/HHMI

**Keywords:** clonal expansion, HIV infection, integration site analysis, perinatal infection

## Abstract

Little is known about the emergence and persistence of human immunodeficiency virus (HIV)-infected T-cell clones in perinatally infected children. We analyzed peripheral blood mononuclear cells (PBMCs) for clonal expansion in 11 children who initiated antiretroviral therapy (ART) between 1.8 and 17.4 months of age and with viremia suppressed for 6 to 9 years. We obtained 8,662 HIV type 1 (HIV-1) integration sites from pre-ART samples and 1,861 sites from on-ART samples. Expanded clones of infected cells were detected pre-ART in 10/11 children. In 8 children, infected cell clones detected pre-ART persisted for 6 to 9 years on ART. A comparison of integration sites in the samples obtained on ART with healthy donor PBMCs infected *ex vivo* showed selection for cells with proviruses integrated in *BACH2* and *STAT5B*. Our analyses indicate that, despite marked differences in T-cell composition and dynamics between children and adults, HIV-infected cell clones are established early in children, persist for up to 9 years on ART, and can be driven by proviral integration in proto-oncogenes.

## Abstract (precis)

HIV-1 integrates its genome into the DNA of host cells. Consequently, HIV-1 genomes are copied with the host cell DNA during cellular division.

## INTRODUCTION

Human immunodeficiency virus (HIV) remains a worldwide health crisis. Approximately 38 million people are living with HIV globally, and about 690,000 people died from AIDS-related illnesses in 2019 (https://www.unaids.org/en/resources/fact-sheet). Although current antiretroviral therapy (ART) is able to fully suppress HIV type 1 (HIV-1) replication in the blood (1–4), lymph nodes (5–8), and other tissues (9, 10), it does not cure the infection. If treatment is initiated before the immune system is heavily compromised and if there is lifelong adherence, ART can lead to a partial restoration of CD4^+^ T-cell numbers (11, 12) and can prevent immunodeficiency in most individuals.

The main obstacle to a cure for HIV-1 is the persistence of replication-competent proviruses in long-lived and/or proliferating populations of infected T cells (13, 14). Most of the infected cells that persist on ART contain defective proviruses that are incapable of producing infectious virus (15, 16). These defective proviruses do not directly contribute to the HIV-1 reservoir that persists on ART but complicate its measurement and may contribute to persistent immune activation. The fraction of infected cells that contains replication-competent (intact/infectious) proviruses has been estimated to be between 1 and 5% in individuals on long-term ART (15–17). In both adults and children, when ART is initiated soon after infection, the number of infected cells is reduced, sometimes to levels below the detection limit of current assays (17–19), and rebound viremia can be significantly delayed (20–23).

Studies of HIV-1 integration sites were initially performed in cell lines and showed that sites were widely distributed but favored highly expressed genes (24–26). Two studies in 2014 were the first to demonstrate expansion of HIV-infected T cells *in vivo* (27, 28). These clones of infected T cells can be detected as early as Fiebig IV in acute infection (29), can persist in adults for at least 3 years on ART (30), and are distributed among different tissues (5). Studies of clones persisting in adults on ART revealed selection against proviruses in expressed genes, with a stronger selection against those that are integrated in the same orientation as the host gene (27; J. M. Coffin, M. J. Bale, D. W. Wells, S. Guo, B. Luke, J. M. Zerbato, M. Sobolewski, T. Sia, W. Shao, W. Xiaolin, F. Maldarelli, M. F. Kearney, J. W. Mellors, and S. H. Hughes, submitted for publication) and selection for proviruses integrated into some proto-oncogenes—e.g., *BACH2*, *MKL2*, and *STAT5B* (27, 28). Although much is now known about the HIV-1 integration site landscape in adults prior to and on ART, there is little information on clonal expansion of infected cells in children who acquired HIV perinatally (PHIV). The largely anti-inflammatory and immunoregulatory environment of the immune systems in children (31, 32) could affect the behavior of infected T cells in ways that would alter the integration site landscape and selection of proviruses at specific sites in children, leading to differences compared to adults. Furthermore, infants have a high fraction of naive T cells and fewer clonally expanded T cells than adults (33, 34), a difference that could affect integration site selection and clonal expansion of infected cells.

To our knowledge, only two reports have investigated clonal expansion of infected cells in children to date (28, 35). However, only a few children were studied, and the integration site sampling in these studies was shallow because it is difficult to collect large numbers of peripheral blood mononuclear cells (PBMCs) from infants and children. One study reported clones of infected cells in 3 children initiating ART during chronic infection (28) and another the same in 3 neonates who were not on ART (35). Here, we expand on these studies to perform a deep look at the integration site landscape in 11 children who initiated ART early and were followed for 6 to 9 years of continual suppression of viremia. We performed a detailed analysis of the integration site landscape by comparing the findings to those in *ex vivo* infected adult PBMCs and to those in infected adults on ART (5, 27). To study the emergence of infected CD4^+^ T-cell clones before ART initiation, the dynamics of their long-term persistence, and their potential survival within select genes, we obtained 10,523 integration sites from CD4^+^ T cells in the perinatally infected children using samples obtained prior to and during long-term ART. We compared longitudinal integration site data sets to look for evidence of long-term persistence of clones of infected T cells and to investigate the frequency and size of the infected cell clones in the children. Finally, to determine if there exists selective maintenance of infected cells within single genes, we analyzed the integration sites in children compared to sites obtained from *ex vivo* infected, CD8-depleted PBMCs (deposited at rid.ncifcrf.gov) (36, 37).

We report here that clones of infected cells are found in children as early as 1.8 months after birth and that some of the clones that arose early persisted for up to 9 years on ART. Strikingly, although there are noted differences between the immune environments in children compared to adults, our findings on the population of infected T-cell clones are similar to what has been reported for adults, suggesting that clonal expansion is the main mechanism for persistence of HIV-1 in children whose viremia is suppressed by ART. We also found that the selection for proviruses integrated in certain genes is similar in adults and children and, importantly, that this selection occurs pre-ART. Integration events and selection for proviruses in these genes in children born with HIV-1 could have long-term effects in adulthood that have not been investigated and that are not observed in adults who were not born with HIV infection.

## RESULTS

### Participants and sampling.

PBMCs were obtained from children enrolled in the Children with HIV and Early Antiretroviral therapy (CHER) randomized trial and post-CHER cohort (38, 39) who were identified as plasma HIV-1 RNA positive by 7 weeks of age, initiated ART within 18 months of age (median, 5.1 months; range, 1.8 to 17.4 months), and had long-term, sustained suppression on ART (2) (see Table S1 in the supplemental materials). Children were included based on the availability of pre-ART PBMCs and PBMCs obtained after at least 6 years of continuous suppression of viremia (median, 8.1 years; range, 6.8 to 9.1 years). The sex, pretreatment plasma HIV-1 RNA, ART regimen, time to viral load suppression, and CD4 percentage after long-term ART are shown in Table S1. The pre-ART and on-ART enchriched-CD4^+^ T cells were analyzed for the presence and persistence of clones of infected cells. We obtained between 197 and 1,386 (median, 655) integration sites from each of the samples taken before ART was initiated and between 77 and 432 (median, 137) integration sites from those after at least 6 years on ART (Table 1). In total, we obtained 10,523 HIV-1 integration sites from the 11 children.

**TABLE 1:** Number of integration sites and infected cell clones detected in children prior to and on ART

Donor ID	Age at ART initiation (mo)	No. of integration sites obtained pre-ART^*a*^	No. of integration sites detected with >2 breakpoints pre-ART (no. with >1 breakpoint)	Oligoclonality index^*b*^ (pre-ART)	Yrs suppressed on-ART	HIV DNA cps/10^6^ PBMC on-ART^*c*^	No. of integration sites obtained on-ART^*a*^	No. of integration sites detected with >1 breakpoint on-ART	Oligoclonality index^*b*^ (on ART)	No. of integration site matches between pre-ART and on-ART^*d*^
ZA002	17.4	1064	27 (50)	0.085	6.87	33	113	9	0.079	7
ZA003	1.8	1386	7 (30)	0.04	8.06	2	148	16	0.161	0
ZA004^*e*^	2.7	655	5 (13)	0.024	7.92/8.76	24/—	255	25	0.223	3
ZA005	6.0	583	2 (14)	0.027	8.04	9	77	4	0.166	1
ZA006	9.0	486	1 (6)	0.012	7.45	47	137	20	0.313	1
ZA007	9.9	197	4 (5)	0.07	8.24	21	85	8	0.403	2
ZA008	2.2	1293	1 (8)	0.006	6.77	42	225	11	0.065	1
ZA009	2.0	809	0 (16)	0.021	9.13	186	125	5	0.055	0
ZA010	1.8	514	1 (12)	0.027	8.35	5	115	3	0.173	0
ZA011	9.3	432	5 (9)	0.037	7.35	182	149	8	0.092	3
ZA012	5.1	1243	3 (12)	0.01	8.41	12	432	32	0.126	2
Median	5.1	655	3 (12)	0.027	8.04	24	137	9	0.161	1

a Value obtained by counting an integration from both the 5′ and 3′ LTRs as a single integration. Site.

b As described in Gillet et al. (42) and Bangham (39). This value ranges in the interval [0, 1] are dependent on the relative size and contribution of integration site clones to the data set, where 0 signifies a completely uniform distribution, and 1 signifies a single integration site.

c Integrase cell-associated DNA (iCAD) protocol (49). —, data not determined.

d Matches between pre-ART and on ART are counted as clones in columns 4 and 9.

e Participant ZA004 had on-ART samples from two separate time points, the first when the individual was suppressed for 7.92 years on ART and the second at 8.76 years.

10.1128/mBio.00568-21.1 TABLE S1 Donor characteristics. Download Table S1, PDF file, 0.1 MB. Copyright © 2021 Bale et al. 2021 Bale et al. https://creativecommons.org/licenses/by/4.0/ This content is distributed under the terms of the Creative Commons Attribution 4.0 International license.

### Clones of HIV-1-infected cells are detected in children pre-ART and persist on long-term ART.

Clonal expansion of cells infected with replication-competent proviruses is an important mechanism for HIV-1 persistence on ART (5, 13, 30, 40, 41). The detection of identical integration sites within a sample is the hallmark of clonal expansion of an infected cell, independent of the replication competence of the integrated provirus. We defined an integration site as being from a clone using three separate criteria, as follows: (i) detection of the same integration site at least 3 times in pre-ART samples (to account for recently infected cells that had duplicated their DNA but would die before establishing a clone), (ii) detection of the same integration site at least twice in an on-ART sample (if a cell is dividing after long-term ART, it is almost certainly part of a clone), and (iii) detection of the same integration site in two different samples from the same donor. Additionally, the method we use to identify integration sites recovers the host-virus DNA junctions from both the 5′ and 3′ long terminal repeats (LTRs) (27). Therefore, integration sites observed at both junctions were considered a single integration site under the conservative assumption that they could have originated from the same provirus. In all but one of the 11 donors (participant identifier [PID] ZA009), we found at least one clone of infected cells in the pre-ART samples (range, 1 to 27) (Table 1, column 4). We found at least 3 clones of infected cells in all on-ART samples (range, 3 to 32) (Table 1, column 9). Although we did not detect any clones of infected cells in the pre-ART samples from donor ZA009 by the stringent criteria described above, 16 of the integration sites were detected twice, suggesting that clones of infected T cells could have been present in this donor pre-ART (Table 1, column 4 parenthetical). We identified clones of infected cells in the pre-ART samples that persisted for up to 6 to 9 years on ART in 8 of the 11 children (range, 1 to 7 clones) (Table 1, column 11). These data show that clonal expansion contributes to the persistence of total HIV-1 DNA in children, as was shown previously for adults (5, 14, 27, 30, 40).

### Size of infected cell clones is similar in children and adults.

**FIG 1:**
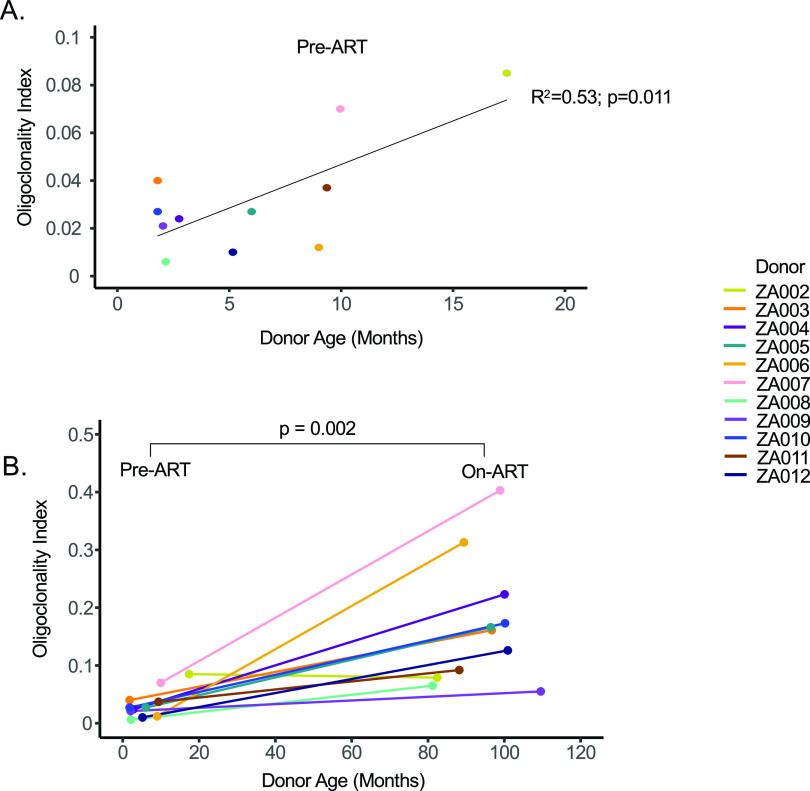
Oligoclonality index (OCI) values correlate with duration of infection prior to ART but not with duration of infection on ART. OCT values were calculated from the pre-ART and on-ART libraries plotted against donor age in months. Pre-ART OCIs were evaluated via linear regression and *F* test against donor age (A), while change in OCI as a function of ART status was evaluated by Wilcoxon signed-rank test (B).

**FIG 2:**
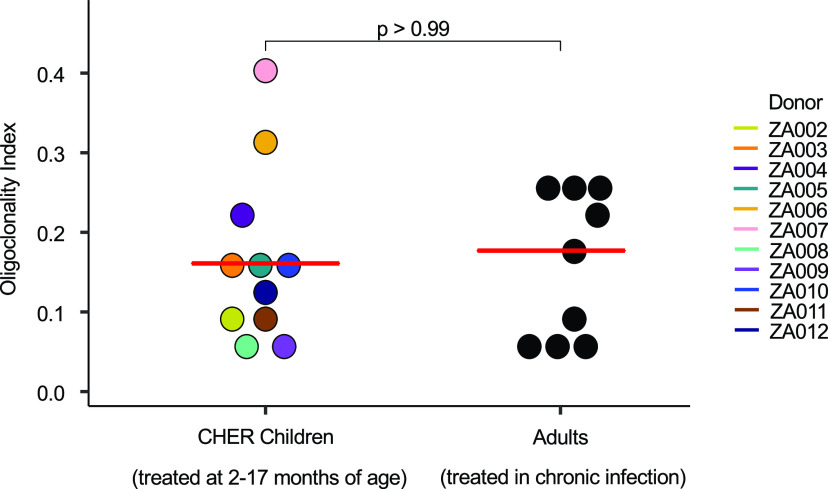
Oligoclonality indexes are comparable between ART-suppressed adults and children. Integration site data from donors whose viremia was suppressed on ART were downloaded from the Retrovirus Integration Database (rid.ncifcrf.gov) (37) from two studies totaling 9 individuals (5, 27) and the OCIs were calculated. OCIs were compared using the Mann-Whitney test. Median values for each patient group are marked by red lines.

We analyzed the size and frequency of infected cell clones using a modified Gini coefficient called the “oligoclonality index” (OCI) (42). Briefly, the OCI, which has a value between 0 and 1, is a measure of the nonuniformity of a given data set; 0 indicates complete heterogeneity, and 1 indicates complete homogeneity. In our analysis, 0 would mean that each detected integration site was detected only once, while a value of 1 would mean that all the integration sites would be from a single large clone. In the pre-ART samples, most integration sites were detected only once (Table 1, column 5). The pre-ART samples contained large numbers of recently infected cells that had not undergone clonal expansion. Thus, all pre-ART OCI values were less than 0.1 (range, 0.006 to 0.085; median, 0.027). The pre-ART OCI positively correlated with the age at which ART was initiated, presumably because clones increase in size with time, which makes it easier for us to detect them (adjusted *R*^2^ = 0.53; *P* = 0.011) (Fig. 1A). Stated differently, although clones can arise soon after infection (30), they may require time to expand to a size that can be detected using the integration site assay (ISA) (30, 35). As expected, the OCI values were significantly higher during long-term suppression on ART (range, 0.055 to 0.403; median, 0.161; *P* = 0.002) (Table 1, column 10, and Fig. 1B), suggesting that the short survival time of most recently infected T cells makes it easier to detect clones of infected cells after long-term ART (27, 30). It should be noted; however, that the on-ART OCI does not correlate with time on ART (adjusted *R*^2^ = −0.08; *P* = 0.63), suggesting that clonal expansion during ART is not just a function of time, but rather a complex dependence on homeostatic, antigen-driven, and integration-driven proliferation (Fig. 1B). We further compared the on-ART OCI in children to published data sets from 9 infected adults (5, 27) on long-term ART and found no statistical difference (*P* > 0.99; Fig. 2; numerical data found in Jupyter Notebook; see Materials and Methods) (43).

### Selection for cells with proviruses integrated into certain genes.

Recent reports show that HIV-1 proviruses integrated into one of a small number of genes (14, 27, 28, 30, 44) contribute to the growth, survival, and persistence of the infected cell clones *in vivo*. To look for evidence of similar selection in children born with HIV-1 and treated early with ART, we compared the distribution of integration sites from the children (pre-ART and on ART) to integration sites obtained from *ex vivo* HIV-1-infected, CD8-depleted PBMCs from healthy donors (deposited at rid.ncifcrf.gov [36]). We asked if there was evidence for enrichment of proviruses in specific genes *in vivo* (relative to *ex vivo*). We also analyzed the orientation of the proviruses relative to the host gene. Enrichment in the fraction of proviruses within, and oriented in the same direction as, the gene are evidence of postintegration selection. Enrichment of the integration sites was determined by comparing the *ex vivo*-infected PBMC data set against the *in vivo* data sets. For this analysis, clonally amplified sites were removed from the *in vivo* data sets by collapsing identical integration sites. Integration sites in intergenic regions (mapped to hg19) were not included in the analysis. The resulting data sets consisted of 335,614 integration sites from the *ex vivo* infected PBMC (87.2% of the initial data), 7,039 sites from the pre-ART data set from the children (83.9%), and 1,202 (76.8%) sites from the on-ART data set from the children. To detect enrichment in both the pre-ART and on-ART data sets relative to the *ex vivo* PBMC data set, Fisher’s exact tests were performed on genes in each library with *post hoc* multiple-test correction. Adjusted *P* values are reported with an adjusted *P* (*P*_adj_) value of ≤0.05 being considered significant.

Consistent with what has been observed in virally suppressed adults (27), we found a strong enrichment for proviruses integrated into both *BACH2* (*P*_adj_ = 2.7 × 10^−15^) and *STAT5B* (*P*_adj_ = 4.0 × 10^−29^) (Table 2), but not *MKL2* (*P*_adj_ > 0.05) during ART. The question of enrichment in samples prior to ART initiation in either adults or children has not previously been addressed. Strikingly, we observed a signal for enrichment of integrations into *BACH2* in children even prior to ART initiation (*P*_adj_ = 8.9 × 10^−17^) showing that selection can occur early in PHIV infection. Although not statistically significant, we also observed a trend toward selection for integration events in *STAT5B* (*P*_adj_ = 0.14) (Table 2) prior to ART initiation.

**TABLE 2:** Analysis of enrichment of integration into specific genes *in vivo*^*a*^

Chromosome	Gene name^*b*^	Independent integrations *ex vivo*^*c*^	Independent integrations in CHER cohort pre-ART	Adjusted *P* value^*d*^	Independent integrations in CHER cohort on ART	Adjusted *P* value^*d*^
17	*STAT5B*	562	29	0.14	37	4.0E−29
6	*BACH2*	132	31	8.9E−17	16	2.7E−15
All	All other genes	334,920	6,979	>0.05	1,149	>0.05

a Data are shown only for integrations into genes and when at least 1 integration was detected in both libraries.

b Genic coordinates mapped to hg19.

c *Ex-vivo* data set contains integration sites from CD8-depeleted PBMCs from two healthy donor patients infected and phytohemagglutinin (PHA)-stimulated *ex vivo*.

d Adjusted *P* value determined by Fisher’s exact test with *post hoc* Benjamini-Hochberg correction.

**FIG 3:**
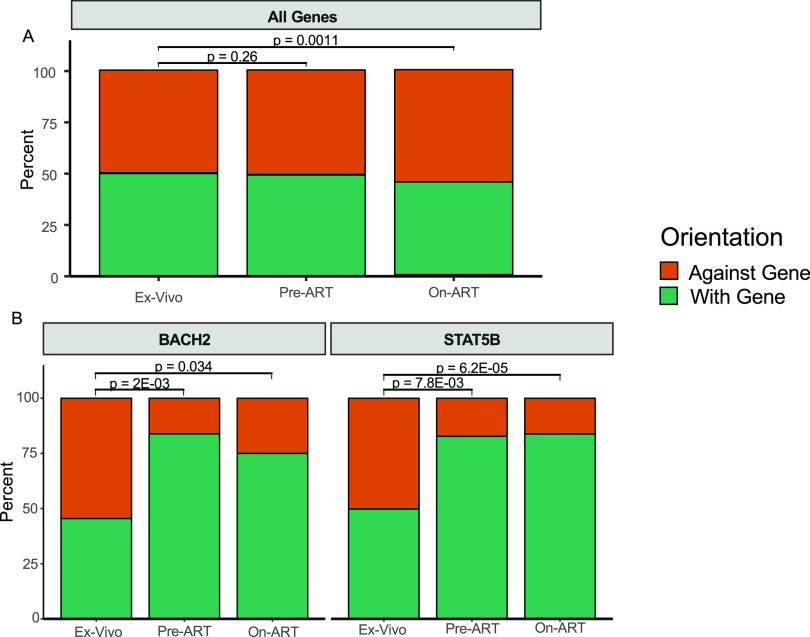
Global selection against proviruses oriented with the gene and selection in two genes for with-the-gene proviruses. For each of the integration site libraries, unique integration sites for all genes (A) and for proviruses integrated into *BACH2* and *STAT5B* (B) were plotted as the percentages of integrations against the gene (orange) and with the gene (green). Significance was assessed via Fisher’s exact test between the *ex vivo* infected PBMC library and the pre-ART and on-ART integration site libraries from children. *P* values for pre-ART comparisons were *post hoc* adjusted. The on-ART comparisons were not adjusted because of the differences in the number of independent statistical tests against each library.

Previous studies in adults have shown that, if there is postintegration selection for an HIV provirus in a gene, like *STAT5B* and *BACH2*, the proviruses are highly enriched for the same orientation as the gene (27, 28). We analyzed the genes for which there were at least 15 unique integrations in the *ex vivo* and *in vivo* data set so that there would be sufficient signal to detect selection. Although 18 genes were retained for analysis in the pre-ART data set, only 2 met these criteria in the on-ART data set (see Tables S2 and S3 in the supplemental material). Despite the global preference for proviruses detected on ART to be integrated against the gene (*ex vivo* PBMCs, 50.0%, versus children on ART, 54.7%; *P* = 0.0011), there was no evidence for such global selection prior to initiation of ART (*ex vivo* PBMCs, 50.0%; children pre-ART, 50.7%; *P* = 0.26 for the difference) (Fig. 3A). However, of the 18 genes in which there were sufficient numbers of integrations in pre-ART samples, we found selection for with-the-gene integration in both *BACH2* (*P*_adj_ = 2.0 × 10^−3^) and *STAT5B* (*P*_adj_ = 7.8 × 10^−3^) (Table S2 and Fig. 3B) and an against-the-gene bias in an ankyrin repeat protein, *ANKRD11* (*P*_adj_ = 0.028) (Table S3). Although these data provide evidence for strong selection for both *BACH2* and *STAT5B* pre-ART, we do not consider the against-gene bias for *ANKRD11* to be evidence of selection specific to that gene because of the global bias for against-gene integrations and the lack of an enrichment signal in this and previous data sets.

10.1128/mBio.00568-21.2 TABLE S2 Orientation bias for genic integrations pre-antiretroviral therapy (ART). Download Table S2, PDF file, 0.1 MB. Copyright © 2021 Bale et al. 2021 Bale et al. https://creativecommons.org/licenses/by/4.0/ This content is distributed under the terms of the Creative Commons Attribution 4.0 International license.

10.1128/mBio.00568-21.3 TABLE S3 Orientation bias for genic integrations on ART. Download Table S3, PDF file, 0.1 MB. Copyright © 2021 Bale et al. 2021 Bale et al. https://creativecommons.org/licenses/by/4.0/ This content is distributed under the terms of the Creative Commons Attribution 4.0 International license.

Likewise, integration sites recovered from children on ART in *BACH2* and *STAT5B* were significantly selected for with-the-gene orientation (*P_BACH2_* = 0.034; *P_STAT5B_* = 6.2 × 10^−5^) (Table S3 and Fig. 3B). Taken together with the enrichment analyses, we conclude that cells containing proviruses integrated in *BACH2* and *STAT5B* in the same orientation as the genes were selected in children both prior to and on ART.

**FIG 4:**
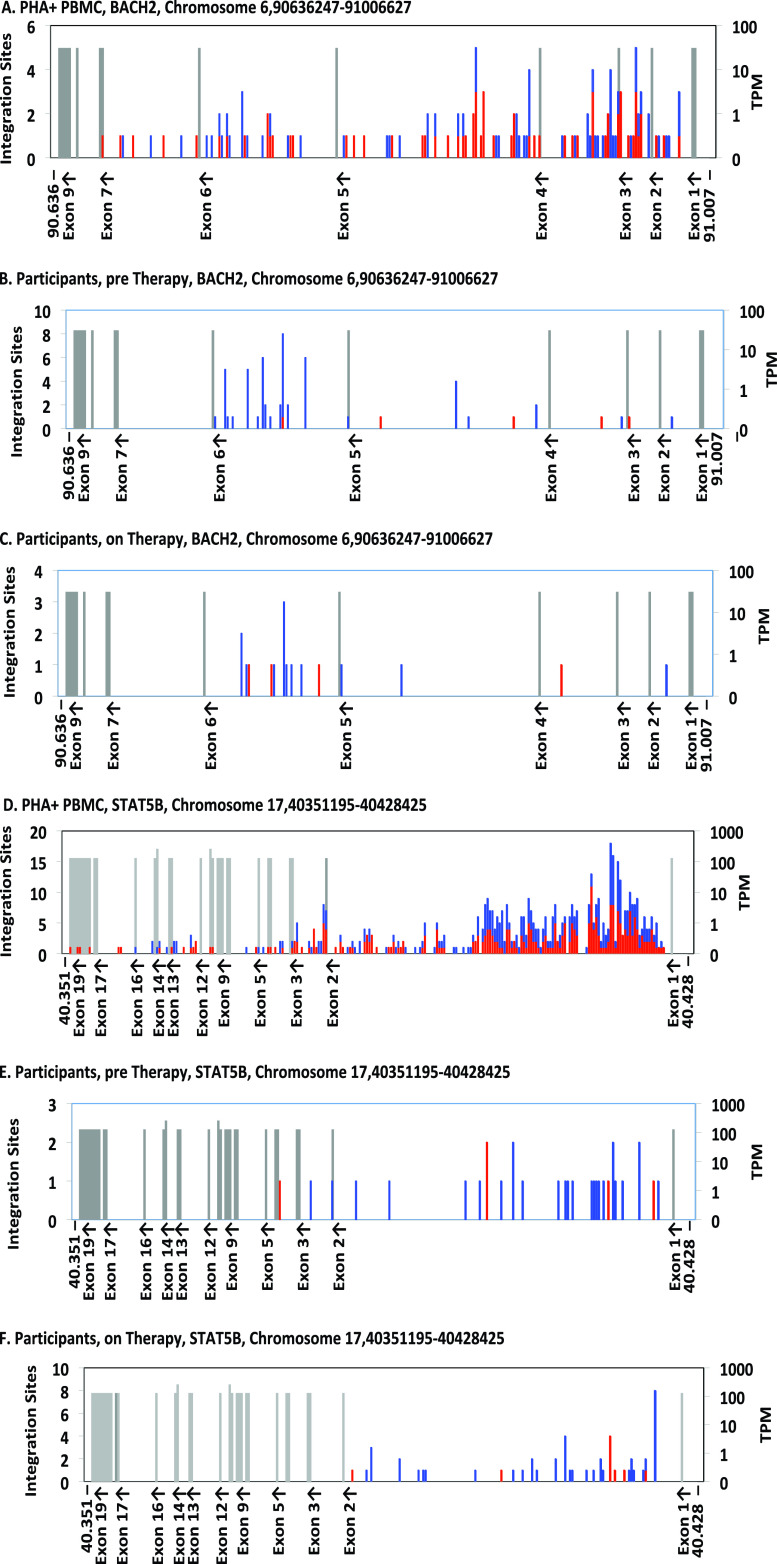
Distribution of integration sites in *BACH2* and *STAT5B.* The maps show the integration sites of the proviruses in *ex vivo* infected PBMCs (A, D) (Coffin et al., submitted), infants sampled pre-ART (B, E), and children sampled on ART (C, F). Each graph shows the entire gene, divided into 250 bins. For *BACH2* (A to C), each bin corresponds to ca. 1,500 nucleotides (nt), and for *STAT5B* (D to F), ca. 300 nt. Exons (labeled on the *x* axis, with orientation of transcription shown) are shown as gray bars whose height indicates the level of expression in transcripts per million (TPM), as shown on the scale on the right. Note that the resolution of the text sometimes leads to loss of labels of closely spaced exons. The numbers of integration sites in each bin are indicated by the stacked bars, according to the scale on the left, with red indicating the same transcriptional orientation as the chromosome numbering and blue indicating the opposite orientation. In these two genes, blue indicates the number of proviruses in each bin integrated in the same orientation as the gene.

We also compared the within-gene distribution of proviruses in *BACH2* and *STAT5B* in the children versus the *ex vivo*-infected PBMCs using an in-house mapping application (Coffin et al., submitted) (Fig. 4). In both genes, clearly visible clusters of integration sites in the same orientation as the gene (shown in blue) in a single intron upstream of the start of translation were observed in the children both before and during ART (Fig. 4B, C, E and F). The *ex vivo*-infected PBMCs have a broader, randomly oriented (equal red to blue) distribution (Fig. 4A and D) in comparison. The different distributions highlight the selection for directional and clustered integration events into *BACH2* and *STAT5B* in children both prior to and on ART.

### Subgenomic sequencing data sets do not accurately characterize clonality within individuals.

**FIG 5:**
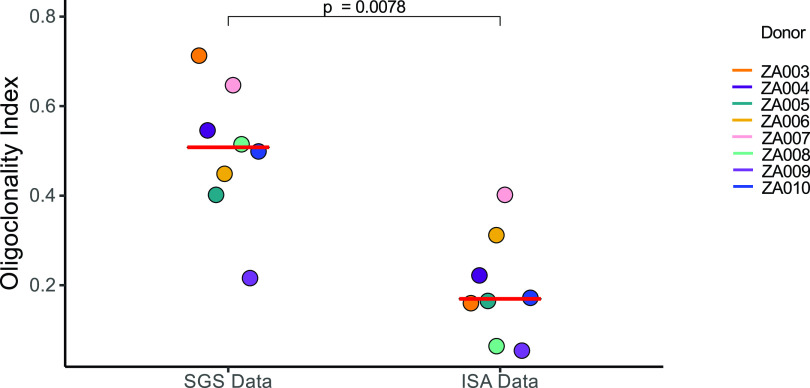
OCI values for single-genome sequencing data sets are significantly higher than OCI values derived from integration sites analyses. OCI values were calculated from single-genome sequencing and integration sites data obtained from PBMCs of children suppressed for 6 to 9 years on ART. Significance was assessed by Wilcoxon signed-rank test. Median values are noted by a red dash for each group.

Proviruses in a subset of the children in this study were previously characterized using single-genome sequencing (SGS) of the *gag-pol* genes (encoding P6, protease, and the first 900 nucleotides of reverse transcriptase [P6-PR-RT]) (2). We assessed the clonality of the infected cells using integration site analysis compared to the identical sequences found in the SGS analysis. We found that proviruses with identical subgenomic sequences were more common and constituted larger fractions of the data than the clones detected by sequencing integration site analyses (Fig. 5; see also Fig. S1 in the supplemental material). We also calculated the OCI for each set of data and found that the OCIs were significantly higher (average fold difference, 3.4×) for the subgenomic single-genome sequences than for the integration site data sets (*P* = 0.0078) (Fig. 5). These data suggest that either proviruses with identical subgenomic sequences have different sites of integration, as has been shown for adults (40), or that many of the integration sites that were detected contained proviruses for which the *gag-pol* regions could not be amplified and sequenced due to deletions, PCR primer mismatches, or both.

10.1128/mBio.00568-21.4 FIG S1 Number of detections of integration sites. For each study participant, a neighbor-joining phylogenetic tree representing *gag-pol* single-genome sequences with its respective oligoclonality index (OCI) value is shown on the left; on the right, a pie chart representing the number of detections of integrations sites by integration site assay (ISA) and the respective OCI value. Download FIG S1, PDF file, 0.2 MB. Copyright © 2021 Bale et al. 2021 Bale et al. https://creativecommons.org/licenses/by/4.0/ This content is distributed under the terms of the Creative Commons Attribution 4.0 International license.

## DISCUSSION

Despite effective therapies which have reduced the rate of mother-to-child HIV-1 transmission (45–47), approximately 150,000 infants were infected worldwide in 2019 (https://www.unaids.org/en/resources/fact-sheet). These children must be included in the larger quest for effective HIV-1 curative interventions, and such interventions may need to be tailored to their developing immune systems. Although the contribution of clonal expansion to HIV-1 persistence is well studied in adults (5, 27, 30), this mechanism has not been well described in children. Additionally, only one analysis has been done in children on the clonal expansion of infected cells prior to the initiation of ART. To compare the mechanisms that underlie the persistence of HIV-1-infected cells during ART in adults and vertically infected children, we performed HIV-1 integration site analysis on samples obtained from perinatally infected infants prior to ART initiation and from the same children during long-term suppression of viremia on ART (6 to 9 years of full suppression on ART). Despite inherent differences in T-cell composition between children and adults (34), the clones of HIV-1 infected cells obtained from the blood of children in our study were not statistically different from those in adults (5, 27).

A study by Coffin et al. showed that infected cell clones can arise in adults in the first few weeks postinfection (30). In this study, we found that infected cell clones were detectable, using the integration sites assay, in 4 of the 5 samples collected from infants of <3 months of age, consistent with early detection of clones in adults (30). In 2 of the 5 infants first sampled at <3 months old, we detected multiple proviruses with identical integration sites in both the pre-ART sample and the 6 to 9 years on ART sample, demonstrating that clones of cells arose prior to ART initiation and persisted for years on ART. The other 6 donors, who were less than 3 months of age when initiating ART, also had detectable infected cell clones that persisted for at least 6 years of treatment. The frequency of clonal detection in the pre-ART populations tracked linearly with the estimated duration of infection prior to ART—using age as a surrogate—suggesting that the number of infected cell clones that expanded to detectable levels increased with the time of untreated infection, at least during the relatively short periods our donors were infected pre-ART. Our finding that infected cell clones had expanded and become large enough to be detected before 2 months of age supports the idea that the HIV-1 reservoir is generated rapidly, in actively dividing cells, in both adults and children (30, 48).

These results, in conjunction with previous studies showing that ongoing HIV-1 replication does not occur in children when viremia is fully suppressed on ART (2, 49, 50) and the fact that intact proviruses persist for years both in adults treated early (15) and in children treated early (17), supports the conclusion that the HIV-1 reservoir is maintained in vertically infected children through the proliferation of cells infected prior to ART initiation, as it is in adults (5, 27, 30, 51). However, the available data are limited by the rarity of infected cells and the very small subset of HIV-infected cells that harbor intact, replication-competent proviruses in children (17).

Although the number of infected cells in children on ART is small, we were able to detect an enrichment in the number and the orientation of proviruses in both *BACH2* and *STAT5B* in the pre-ART and on-ART samples, suggesting that proviruses in a specific intron and oriented with these genes can promote the survival of these clones *in vivo*, as in adults (27). While the selection for the survival of cells harboring *BACH2* and *STAT5B* proviruses has been previously described in adults on ART (27, 28), no data had been presented to show that such selection exists prior to ART initiation. In both pre-ART and on-ART samples, we saw clear evidence for selection of cells containing proviruses in the exon immediately upstream of the start site of translation in *BACH2* and *STAT5B*. Although the selection of *BACH2* integrants pre-ART was largely driven by a single child (ZA002) who did not initiate ART until 17 months of age, this single example nonetheless shows that clonal selection due to integration in specific genes is not strictly an on-ART phenomenon. The duration of untreated infection in this child may have allowed enough time for the selection of the cells with the *BACH2* proviruses to become detectable. Similar conclusions can be drawn for selection for proviruses integrated into *STAT5B*, where there was clear evidence of selection for cells containing proviruses in the first intron, despite it being a very strong target for integration *ex vivo*. The trend toward enrichment of *STAT5B* integrants in pre-ART samples was due to the high level of sampling required to overcome the background of integration events in this gene compared to the *ex vivo* infected PBMC data set; however, the statistically significant orientation bias prior to ART demonstrates that pre-ART selection exists for *STAT5B*.

The children in the CHER trial who were treated earliest initiated ART at 1.8 months of age, relatively “early” considering the age of the cohort (15 years) and the treatment guidelines in South Africa at the time (39). However, present-day guidelines recommend initiating ART at birth for high-risk infants, significantly reducing the duration of untreated infection (52). In one such cohort, Garcia-Broncano et al. obtained ∼30 integration sites from PBMCs collected 7 h after birth from three infants (35). Their study found one integration site twice (a possible cell clone) but did not show an enrichment for integrations in *BACH2* or *STAT5B*. These data suggest that the occurrence of such clones may be preventable with early treatment; however, sampling did not provide for the opportunity to query for clonal expansions on ART.

Samples from a subset of the children studied here were previously characterized in experiments that showed that ART is effective in suppressing ongoing cycles of viral replication in children (2). Thus, proviral SGS data were available at the same on-ART time point. The OCI values obtained using the P6-PR-RT SGS results were significantly higher than the OCIs obtained from the on-ART integration site data. The observation that a higher OCI was obtained from the SGS data than from the ISA data adds to the growing number of studies (14, 35, 40) suggesting that viruses with identical subgenomic sequences may not all come from a clonal population of infected cells. These data strongly suggest that subgenomic sequencing does not always accurately identify clones of infected cells or sufficiently characterize the genetic diversity of the intrapatient HIV-1 populations that persist on ART (40). Although the results here are consistent with previous studies showing that subgenomic sequences are not sufficient to define clonality, it should be noted that calculating an OCI for small-*N* data sets can result in artificially high OCI values. Studies that are based on integration site analysis, rather than SGS, are more appropriate to study the clonal expansion of infected cells.

There are some caveats to our study. First, it is important to note that because these children were diagnosed within a few weeks of birth, it is not known whether the transmission of HIV-1 occurred at birth or *in utero*. Because of this ambiguity, the age of the participant may not accurately reflect the duration of infection, although we found evidence of clonal expansion as early as 1.8 months after birth. Second, several recent studies have shown that the naive CD4^+^ T-cell compartment can harbor equal or higher levels of inducible and intact HIV compared to other cell subsets in treated individuals (53–56). However, due to the limited sample, we were unable to assess the distribution of infected clonal populations in the various CD4^+^ T-cell subsets in these children. Therefore, it is possible that there are differences in the populations and expansions of infected T cells that we could not detect in the bulk CD4^+^ T-cell populations that we evaluated. Furthermore, the integration site libraries only represent a small fraction of the total number of infected cells in the blood. It is therefore likely that many of the integration sites that were recovered only once belong to clones of infected cells.

Despite these caveats, we have presented here the largest data set yet of integration sites from pediatric HIV-1 infections both prior to and after durable suppression on ART. Because children primarily have naive T cells, which express low levels of the HIV coreceptor CCR5 as a surface marker (34), as well as an immune environment that promotes quiescence (31, 32) and a more diverse T-cell receptor repertoire (33), it is important to determine if there are differences between the observed frequency of clones and patterns of integration and postinsertional selection in children and adults. However, despite the differences in the immune systems of adults and children, our data suggest that these differences do not influence the infection and clonal expansion of T cells to a degree that is detectable by our integration site analysis. It is possible that by 6 to 9 years of age the immune system may be similar enough to that of an adult to account for the striking similarities in the on-ART libraries of these children and the published data from adults. Although these data suggest that the role of clonal expansion as the mechanism for HIV-1 persistence during ART is similar in children and adults, further studies are warranted to better understand how the developing immune system affects clonal expansion and what effects proposed curative interventions might have in both children and adults.

## MATERIALS AND METHODS

### Study approval and ethics statement.

The CHER trial is registered with ClinicalTrials.gov (registration no. NCT00102960). Guardians of all donors provided written informed consent, and the study was approved by the Stellenbosch University Internal Review Board.

### Total HIV-1 DNA quantification.

HIV-1 DNA levels were determined using the integrase cell-associated DNA (iCAD) assay as previously described (57) with the following primers for use with HIV-1 subtype C:

Forward primer HIV_Int_FP CCCTACAATCCCCAAAGTCA 4653 → 4672

Reverse primer HIV_Int_RP CACAATCATCACCTGCCATC 5051 → 5070

### Isolation and purification of cryopreserved CD4^+^ T cells.

Total CD4^+^ T cells were isolated and purified from cryopreserved PBMCs by positive selection using the EasySep cell separation technology (Stemcell Technologies, Vancouver, Canada). CD4^+^ T-cell isolations were performed on on-ART samples, as the children were older and larger blood volume collection was feasible. Briefly, cells were thawed, pelleted, and resuspended in EasySep buffer to a concentration of 50 × 10^6^ cells/ml. The EasySep total CD4^+^ T-cell enrichment cocktail was added to the cells, followed by the addition of magnetic particles. The mixture was then incubated on the Easy 50 EasySep magnet for 10 min at room temperature. The CD4^+^ T-cell enriched cell suspension was then pelleted and resuspended in phosphate-buffered saline (PBS) before sample extraction.

### Integration site assay.

An integration site assay (ISA) was performed and analyzed as previously described (27, 58) using patient-specific primers to the 5′ and 3′ LTRs. Importantly, our protocol includes a shearing step (59) that effectively tags each DNA molecule, allowing determination of the relative numbers of cells in the initial pool with identical sites of integration (i.e., clonality). The full set of integration sites obtained has been submitted to the Retroviral Integration Sites Database (https://rid.ncifcrf.gov/) (37), and the primer sequences are available in Table S5 in the supplemental material.

A comparison integration site data set was prepared from CD8-depleted PBMCs isolated from two HIV-negative human donors infected *in vitro* with replication-competent HIV-1 subtype B (BAL) (60). After 2 days, the cells were harvested and DNA was prepared and integration sites analyzed as described elsewhere (Coffin et al., submitted). The global distribution of the integration sites from the two donors, was indistinguishable; therefore, all comparisons were performed with combined data from the two donors.

### Oligoclonality index.

The oligoclonality index (OCI) was calculated using a python script available at https://github.com/michaelbale/python_stuff/. Full details of the calculation are described in the supplemental text of Gillet et al. (42). Briefly, the LTR-corrected counts of all unique integration sites are sorted into descending order and the cumulative abundances of the clones are summed as a fraction of the total number of unique integration sites and normalized to have a maximal value of 1. Mathematically, the OCI is calculated as below:

*s_i_*: LTR-corrected count of integration site *i*

*S*: Number of unique integration sites in library

$N={\text{Σ}}_{i=1}^{S}s_{i}:$ Total number of integration sites in library

$p_{i}=\frac{s_{i}}{N}:$ Relative abundance of integration site *i*

$X_{i}={\text{Σ}}_{k=1}^{i}p_{k}:$ Cumulative abundance of all integration sites of size {*s_i_*} or greater

$\text{OCI}=2\times ({\text{Σ}}_{k=1}^{S}\left(\frac{X_{k}}{S}\right)-0.5):$ Oligoclonality index of library

### Statistical analysis.

Clonality was assessed by grouping sequenced integration sites with identical pseudo-3′ LTR genomic coordinates and different shear points into count data in R. These count data were used to generate the OCI. Independent integration sites into genes were pooled and assessed for selection by Fisher’s exact test by either the pre-ART or on-ART library versus the *ex vivo* infected PBMC library as a null set. *P* values for this gene enrichment analysis were corrected *post hoc* by the Benjamini-Hochberg method. Orientation biases were assessed in a similar manner with *post hoc* corrections in the pre-ART comparison only. All adjusted *P* values are presented as *P*_adj_ where appropriate. All other statistical analyses are noted where appropriate and were performed in R v3.5.2. A Jupyter notebook (43) with the R commands and visualizations for the unedited figures available at github.com/michaelbale/cher_bale/.

### Phylogenetic analyses.

HIV-1 P6-PR-RT sequences were aligned to HIV consensus C using MUSCLE, and neighbor-joining phylogenetic p-distance trees were built using MEGA 7 (https://www.megasoftware.net/) (61) and outgroup rooted to consensus C. Distance matrix generation for calculation of the sequence-based OCI was performed using Hamming distance.

### Data availability.

Previously published sequences from Van Zyl et al. (2) were accessed from GenBank (accession numbers KY820119 to KY820376), retaining only the sequences associated with the on-ART time point of PIDs ZA003 to ZA010. The integration site libraries for the donors in this study are available in the Retrovirus Integration Database (RID) (37) at rid.ncifcrf.gov. The *ex vivo* infected PBMC integration site library is also available in the RID under PubMed identifier 31291371 (deposited at rid.ncifcrf.gov) (36).
