# Supplementary material for: Early Emergence and Long-Term Persistence of HIV-Infected T-Cell Clones in Children
Source: mBio. 2021 Apr 8;12(2):e00568-21. doi: 10.1128/mBio.00568-21 (PMC8092253; doi:10.1128/mBio.00568-21)
Supplement: TABLE S1 [file mBio.00568-21-st001.pdf]

Table S1. Donor Characteristics.

PID	Sex	Time to viral load suppression in years	Pre-ART plasma HIV RNA[a]	ART regimen[b]	Years suppressed on ART	CD4% at on-ART time point
ZA002	Male	1.37	654000	AZT/3TC/LPV/r	6.87	33
ZA003	Male	0.46	>750000	ABC/3TC/LPV/r	8.06	21
ZA004[c]	Male	1.38	>750000	AZT/3TC/LPV/r	7.92/8.76	46
ZA005	Male	0.47	>750000	AZT/3TC/LPV/r	8.04	41
ZA006	Female	0.44	635000	AZT/3TC/LPV/r	7.45	50
ZA007	Male	0.92	>750000	AZT/3TC/EFV	8.24	35
ZA008	Female	0.44	>750000	AZT/3TC/LPV/r	6.77	36
ZA009	Female	3.76	>750000	AZT/3TC/EFV	9.13	29
ZA010	Female	0.46	510000	AZT/3TC/LPV/r	8.35	39
ZA011	Female	2.29	>750000	AZT/3TC/LPV/r	7.35	54
ZA012	Male	0.93	277,000	AZT/3TC/LPV/r	8.41	31

[a]Determined by Roche Amplicor HIV Monitor assay v1.0

[b]ART abbreviations: Zidovudine (AZT), Lamivudine (3TC), Ritonavir-boosted Lopinavir (LPV/r), Efavirenz (EFV)

[c] Participant ZA004 had on-ART samples from two separate time-points, the first when the individual was suppressed for 7.92 years on ART and the second for 8.76 years.
