# Supplementary material for: Early Emergence and Long-Term Persistence of HIV-Infected T-Cell Clones in Children
Source: mBio. 2021 Apr 8;12(2):e00568-21. doi: 10.1128/mBio.00568-21 (PMC8092253; doi:10.1128/mBio.00568-21)
Supplement: TABLE S2 [file mBio.00568-21-st002.pdf]

Table S2. Orientation Bias for Genic Integrations Pre-ART[a].

Chromosome	Gene name[b]	Unique integrations with the gene (CHER)[c]	Unique integrations against the gene (CHER)[c]	Unique integrations with the gene (*ex vivo*)[c]	Unique integrations against the gene *(ex vivo)*[c]	Adjusted p-value[d]
chr6	*BACH2*	26	5	60	72	0.0019
chr17	*STAT5B*	24	5	284	278	0.0078
chr16	*ANKRD11*	3	16	380	403	0.0281
chr22	*HORMAD2*	11	4	192	241	0.15
chrX	*MECP2*	4	12	228	267	0.45
chr17	*VMP1*	6	12	376	374	0.52
chr17	*GRB2*	11	5	339	336	0.52
chr17	*NPLOC4*	5	10	418	408	0.52
chr17	*POLR2A*	6	10	161	177	0.82
chr11	*MALAT1*	6	9	113	96	0.82
chr11	*PACS1*	18	21	852	821	0.84
chr11	*KDM2A*	9	10	806	724	0.84
chr19	*CARD8*	10	7	331	315	0.84
chr19	*VAV1*	7	8	255	222	0.84
chr17	*RPTOR*	14	12	820	807	0.89
chr17	*CYTH1*	10	9	352	363	0.89
chr22	*TNRC6B*	9	10	444	435	0.89
chr1	*ASH1L*	8	9	378	385	>0.99

a Data shown only for integrations into genes for which at least 15 unique integrations were detected

b Genic coordinates mapped to hg19

c "With" gene and "Against" gene defined as orientation of integrated provirus compared with the sense of the host gene it's integrated into

d Adjusted p-value determined by Fisher Test with post-hoc Benjamini-Hochberg Correction
