# Supplementary material for: Early Emergence and Long-Term Persistence of HIV-Infected T-Cell Clones in Children
Source: mBio. 2021 Apr 8;12(2):e00568-21. doi: 10.1128/mBio.00568-21 (PMC8092253; doi:10.1128/mBio.00568-21)
Supplement: TABLE S3 [file mBio.00568-21-st003.pdf]

Table S3. Orientation Bias for Genic Integrations On-ART[a].

Chromosome	Gene name[b]	Unique integrations with the gene (CHER) [c]	Unique integrations against the gene (CHER) [c]	Unique integrations with the gene (*ex vivo*) [c]	Unique integrations against the gene *(ex vivo)* [c]	p-value[d]
chr17	*STAT5B*	31	6	284	278	6.2E-05
chr6	*BACH2*	12	4	60	72	0.034

[a] Data shown only for integrations into genes for which at least 15 unique integrations were detected in vivo and at least 1 unique integration ex vivo

[b] Genic coordinates mapped to hg19

[c] "With" gene and "Against" gene defined as orientation of integrated provirus compared with the sense of the host gene it's integrated into

[d] p-Value determined by Fisher Test – no post-hoc adjustments performed
