# Supplementary material for: Early Emergence and Long-Term Persistence of HIV-Infected T-Cell Clones in Children
Source: mBio. 2021 Apr 8;12(2):e00568-21. doi: 10.1128/mBio.00568-21 (PMC8092253; doi:10.1128/mBio.00568-21)
Supplement: FIG S1 [file mBio.00568-21-sf001.pdf]

Figure S1. Number of Detections of Integration Sites.

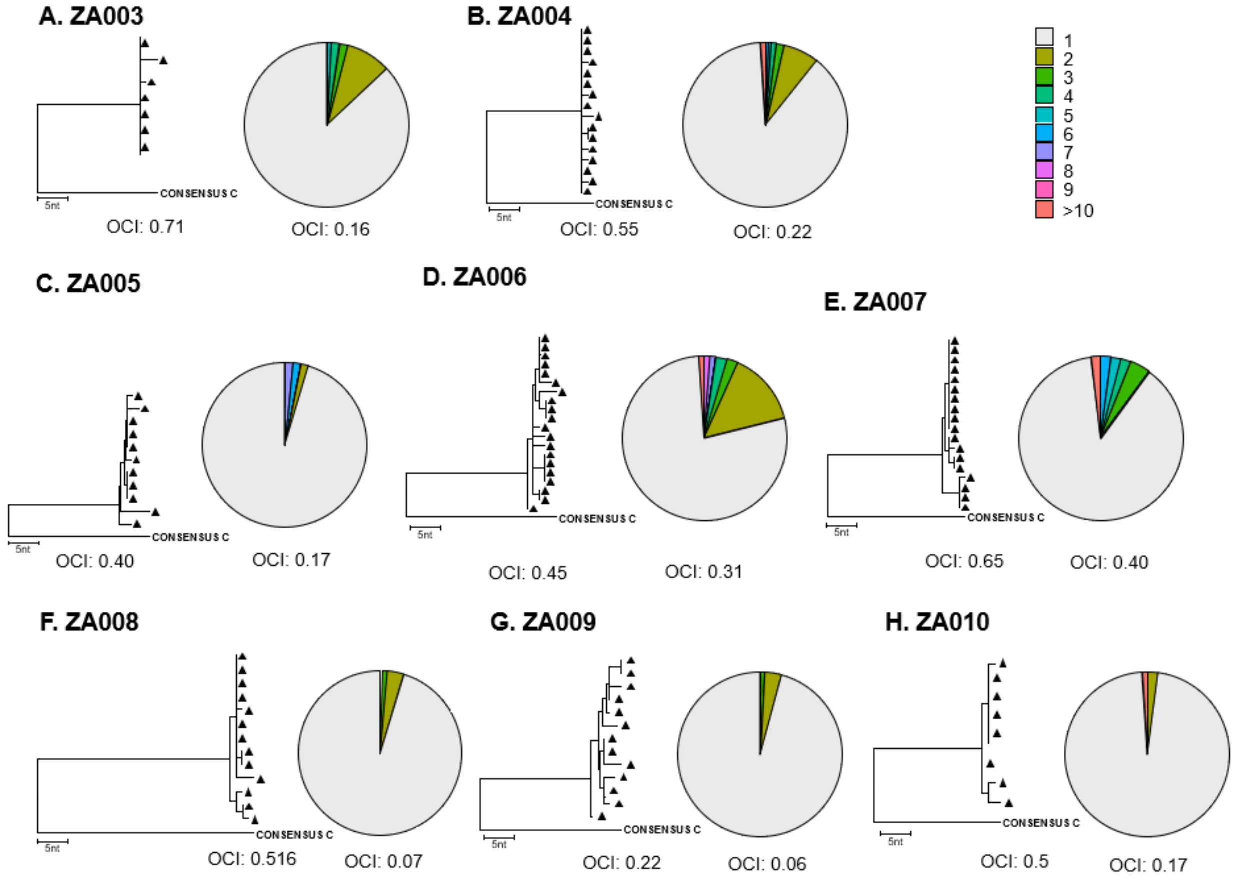

For each study participant, a neighbor-joining phylogenetic tree representing *gag-pol* single genome sequences with its respective OCI value is shown on the left; on the right, a pie chart representing the number of detections of integrations sites by ISA and the respective OCI value.
